# N-terminal acetylation controls multiple functional aspects of the influenza A virus ribonuclease PA-X

**DOI:** 10.1128/jvi.01999-25

**Published:** 2026-01-09

**Authors:** Raecliffe E. Daly, Cynthia Y. Feng, Charles R. Hesser, Idalia Myasnikov, Marta Maria Gaglia

**Affiliations:** 1 Program in Cellular, Molecular and Developmental Biology, Tufts University Graduate School of Biomedical Sciences 50910, Boston, Massachusetts, USA; 2 Institute for Molecular Virology and Department of Medical Microbiology and Immunology, University of Wisconsin-Madison 732057, Madison, Wisconsin, USA; 3 Microbiology Doctoral Training Program, University of Wisconsin-Madison 5228, Madison, Wisconsin, USA; University Medical Center Freiburg, Freiburg, Germany

**Keywords:** N-terminal acetylation, host shutoff, PA-X, influenza A virus

## Abstract

To counteract host antiviral responses, influenza A virus triggers a global reduction of cellular gene expression, a process termed “host shutoff.” A key effector of influenza A virus host shutoff is the viral endoribonuclease PA-X, which degrades host mRNAs. While many of the molecular determinants of PA-X activity remain unknown, a previous study found that N-terminal acetylation of PA-X is required for its host shutoff activity upon ectopic expression. However, it remains unclear how this co-translational modification promotes PA-X activity. Here, we report that PA-X N-terminal acetylation has two functions—it promotes nuclear localization but is also needed directly for host shutoff. Moreover, these two functions can be separated based on whether acetylation occurs on the first amino acid, the initiator methionine, or the second amino acid following initiator methionine excision. Modification at either site is sufficient to ensure PA-X localization to the nucleus, whereas N-terminal acetylation of the initiator methionine is specifically required for normal PA-X host shutoff activity. We also demonstrate that PA-X N-terminal acetylation is needed for its activity during infection. Our studies thus uncover a multifaceted role for PA-X N-terminal acetylation in the regulation of this important immunomodulatory factor.

**IMPORTANCE:**

Influenza A viruses pose a significant threat to human health through seasonal epidemics and recurrent pandemics. Our immune and inflammatory responses have a key role in disease outcome. They clear the virus but can also cause lung damage. Influenza A viruses encode factors that modulate these responses, including PA-X, which destroys cellular mRNAs to control immune responses (a phenomenon called “host shutoff”). PA-X is modified with an acetylation at its N-terminus. This modification is needed for its activity, but it has remained unclear why. We show that PA-X N-terminal acetylation ensures that PA-X goes to the nucleus but also separately contributes to host shutoff activity. For host shutoff activity, the specific location of the modification matters, whereas for entry into the nucleus, it does not. These findings uncover how influenza A viruses exploit a widespread protein modification to support the activity of one of their important immunomodulatory proteins.

## INTRODUCTION

Although influenza A viruses have a relatively small genome of approximately 13.5 kb, multiple influenza proteins are involved in modulating the host immune response. Of the influenza immunomodulatory proteins identified to date, PA-X has emerged as a key factor in regulating gene expression and innate immune responses. PA-X is an accessory protein encoded on segment 3 of the influenza A genome (1), which codes for the polymerase acidic (PA) subunit of the viral RNA polymerase, through ribosomal frameshifting (1, 2). Sequence analysis indicates that all influenza A virus strains encode PA-X (1, 2). Studies using various influenza A strains engineered to lack PA-X have revealed that by limiting innate immune and inflammatory responses during infection, PA-X reduces inflammation-induced pathology (1, 3). Immune regulation by PA-X is the result of its crucial role in influenza host shutoff, that is, the global reduction of gene expression during infection (1, 4–6). However, since PA-X was only discovered in 2012 (1), studies of this protein are still limited.

Previous studies have focused on how PA-X drives host shutoff and have shown that PA-X has endoribonuclease (endoRNase) activity (1, 5–7). PA-X fragments host mRNAs, while largely sparing viral RNAs (5, 6). PA-X discriminates host and viral mRNAs in part based on its sequence preference (7), as it preferentially cleaves GCUG tetramers within hairpin loops, which are enriched in the human but not the influenza transcriptome (7). PA-X also preferentially degrades mRNAs that are spliced, whereas most viral mRNAs are not (6). While these previous studies have dissected the specificity of RNA targeting by PA-X, we still have limited information on how PA-X functions. We and others have shown that it accumulates in the nucleus, and that nuclear localization is required for activity (5, 8). We also previously reported that PA-X is rapidly turned over, with protein half-lives ranging from ~30 min to ~3.5 h depending on the strain (9). Whether and how PA-X activity is modulated in other ways in cells remains unclear.

**Fig 1:**
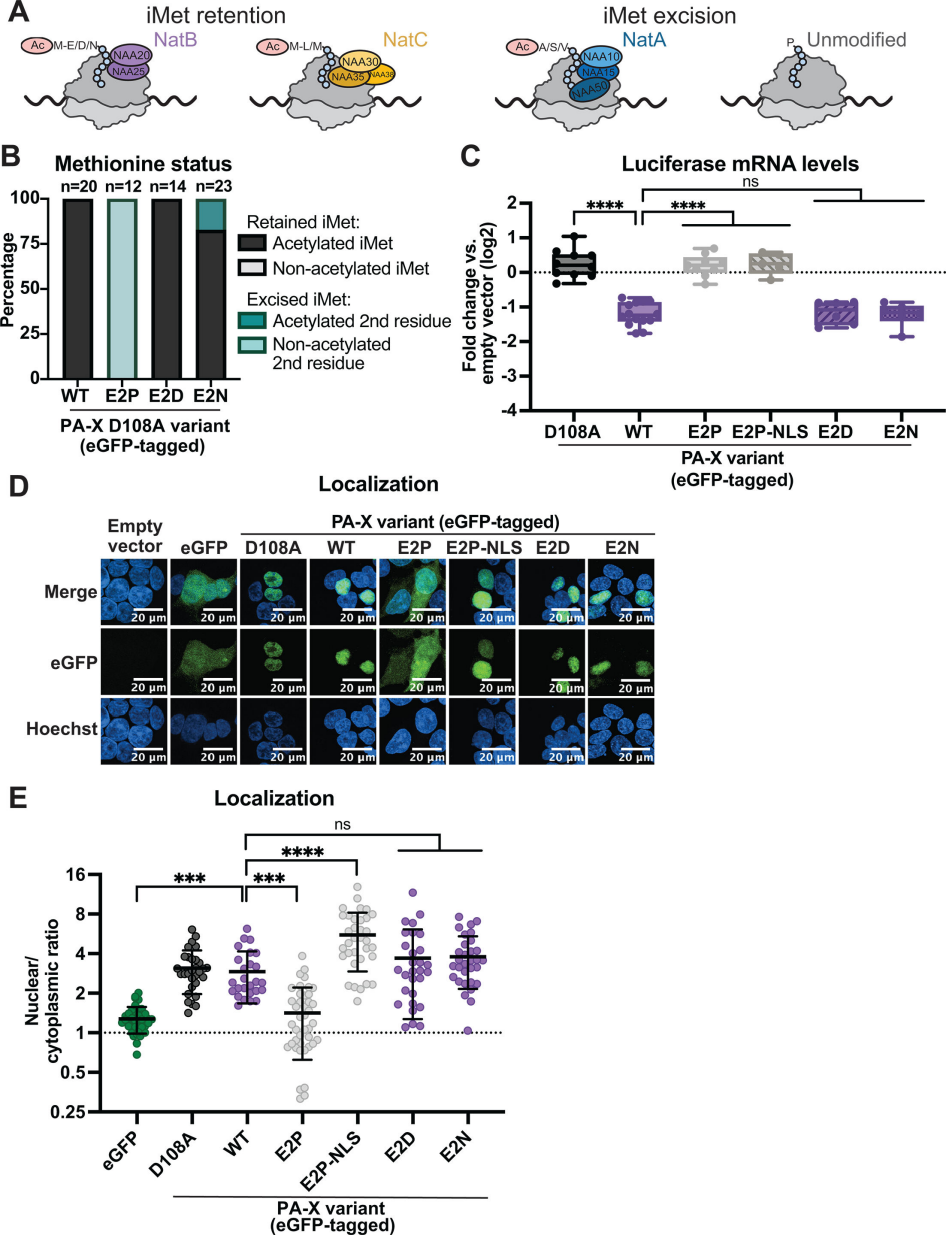
N-terminal acetylation of PA-X is required for nuclear localization. (**A**) Diagram showing NatA-C complexes and the proteins they acetylate depending on the first two amino acids, and whether they require excision of the initiator methionine (iMet). Based on the described properties of NatA-C, WT PR8 PA-X and the PR8 PA-X E2 mutants E2D and E2N should be modified by NatB (purple), PA-X E2L and E2M mutants should be modified by NatC (yellow), and PA-X E2A, E2S, and E2V mutants should be modified by NatA (blue). The PA-X E2P mutant should be unmodified. (**B–E**) HEK293T cells were transfected for 24 h with empty vector, WT PR8 PA-X-eGFP, the catalytically inactive PA-X D108A-eGFP mutant, the indicated PR8 PA-X-eGFP mutants carrying changes in the second amino acid in the WT or D108A PR8-PA-X-eGFP background, or an eGFP-tagged PR8 PA-X E2P mutant carrying an SV40 nuclear localization sequence (NLS; PA-X E2P-NLS-eGFP). (**B**) eGFP-tagged proteins were isolated by immunoprecipitation, and peptides were analyzed by nanoLC-MS/MS for iMet retention and N-terminal acetylation. For these experiments, catalytically inactive versions of PA-X were used, and “WT” means WT sequence at aa 2. N above stacked bars indicates total detected peptides for each variant over two experiments. To note, no non-acetylated iMet was detected in any of the samples plotted in this figure. (**C**) mRNA levels of a co-transfected luciferase reporter were measured by RT-qPCR and normalized to 18S rRNA. Levels are plotted as fold change relative to vector-transfected cells in log2 scale. (**D**) Confocal microscopy was used to image the GFP signal in transfected cells. Nuclei were stained with Hoechst. (**E**) Nuclear/cytoplasmic ratios were calculated by measuring the mean fluorescence intensity of the nuclear and cytoplasmic compartments using ImageJ. Hoechst staining was used as a guide to generate regions of interest for the nucleus. Autofluorescence was used as a guide to generate cytoplasmic regions of interest. Nuclear/cytoplasmic ratios represent the following localization: <1: protein is primarily cytoplasmic, ~1: protein is diffuse throughout the cell, >1: protein is primarily nuclear. For all experiments, some conditions were carried out in parallel to the experiments in Fig. 2 and 3. Therefore, control conditions (empty vector, PA-X D108A, WT PA-X, and eGFP) for the RT-qPCR, mass spectrometry, and microscopy data and quantitation are identical, including representative images for empty vector, eGFP, and WT PA-X. *N*≥3 for all experiments except the mass spectrometry analysis in panel A. For microscopy, representative images are shown. In nuclear/cytoplasmic ratio quantitation, each point represents 1–4 eGFP-positive cells, with 10–15 eGFP-positive cells quantified per condition in each biological replicate. ns = *P* > 0.05, ***P < 0.001, ****P < 0.0001, ANOVA with Tukey’s multiple comparison test.

To date, only one protein modification of PA-X has been reported. Oishi et al. found that PA-X is N-terminally acetylated by the host N-terminal acetyltransferase complex B (NatB) (10). N-terminal acetylation is a highly abundant co-translational protein modification in cells, present on 80%–90% of the human proteome (11–14). It is catalyzed by several Nat complexes (NatA-NatF), which differ in their subunit composition and substrate specificity (12, 14). NatA, NatB, and NatC are responsible for the majority of N-terminal acetylation events in eukaryotes (12, 14). Their substrate specificity is mainly determined by the identity of the second N-terminal residue of the target protein (12, 14) (Fig. 1A). NatB and NatC acetylate nascent polypeptides on the very first translated amino acid, the initiator methionine, whereas NatA modifications occur following the excision of the initiator methionine by methionine aminopeptidases. PA-X starts with an ME- sequence that is highly conserved among influenza isolates (10). Because proteins beginning in ME- are acetylated by the NatB complex with almost 100% efficiency (11, 15), almost all natural variants of PA-X are expected to be acetylated at a high percentage by NatB (10). Despite the fact that N-terminal acetylation is a common modification, there are limited studies on its effects on protein function (11, 12, 16). Nonetheless, N-terminal acetylation has been implicated in multiple processes, including the control of protein stability (17–25), protein folding (26–28), protein-protein interactions (29–34), and protein targeting to membranes, particularly the ER and Golgi (35–38). While Oishi et al. showed that PA-X requires N-terminal acetylation to carry out host shutoff (10), it remains unknown how this modification supports PA-X host shutoff activity.

**Fig 2:**
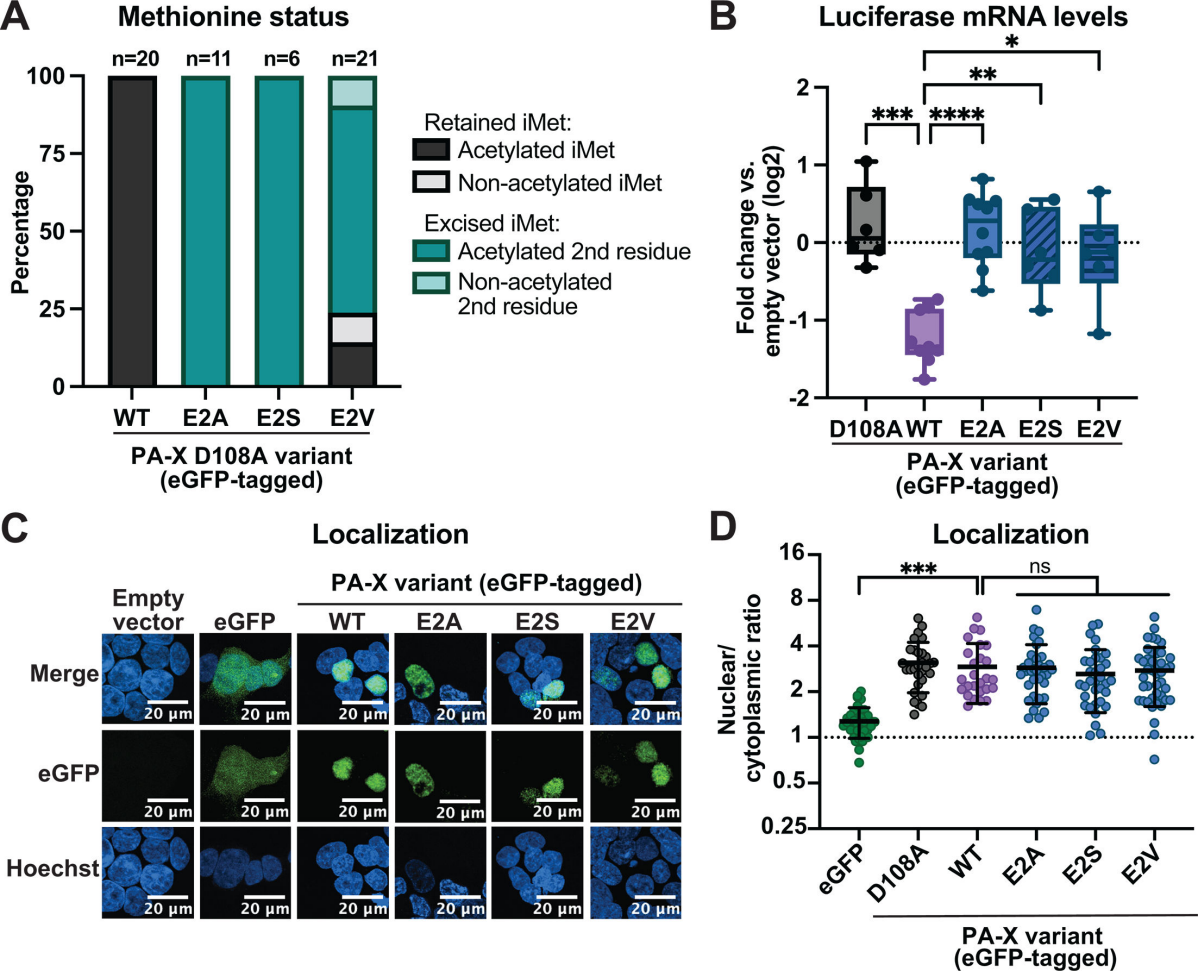
General N-terminal acetylation is sufficient for PA-X localization to the nucleus. HEK293T cells were transfected for 24 h with empty vector, WT PR8 PA-X-eGFP, the catalytically inactive PA-X D108A-eGFP mutant, or the indicated NatA-modified mutants carrying changes in the second amino acid in the WT or D108A PR8-PA-X-eGFP background. (**A**) eGFP-tagged proteins were isolated by immunoprecipitation, and peptides were analyzed by nanoLC-MS/MS for initiator methionine (iMet) retention and N-terminal acetylation. For these experiments, catalytically inactive versions of PA-X were used, and “WT” means WT sequence at aa 2. N above stacked bars indicate total detected peptides for each variant over two experiments. (**B**) mRNA levels of a co-transfected luciferase reporter were measured by RT-qPCR and normalized to 18S rRNA. Levels are plotted as fold change relative to vector-transfected cells in log2 scale. (**C**) Confocal microscopy was used to image the GFP signal in transfected cells. Nuclei were stained with Hoechst. (**D**) Nuclear/cytoplasmic ratios were calculated by measuring the mean fluorescence intensity of the nuclear and cytoplasmic compartments using ImageJ. Hoechst staining was used as a guide to generate regions of interest for the nucleus. Autofluorescence was used as a guide to generate cytoplasmic regions of interest. Nuclear/cytoplasmic ratios represent the following localization: <1: protein is primarily cytoplasmic, ~1: protein is diffuse throughout the cell, >1: protein is primarily nuclear. For all experiments, some conditions were carried out in parallel to the experiments in Fig. 1 and 3. Therefore, control conditions (empty vector, PA-X D108A, WT PA-X, and eGFP) for the RT-qPCR, mass spectrometry, and microscopy data and quantitation are identical, including representative images for empty vector, eGFP, and WT PA-X. *N* ≥ 3 for all experiments except the mass spectrometry analysis in panel A. For microscopy, representative images are shown. In nuclear/cytoplasmic ratio quantitation, each point represents 1–4 eGFP-positive cells, with 10–15 eGFP-positive cells quantified per condition in each biological replicate. ns = *P* > 0.05, *P < 0.05, **P < 0.01, ***P < 0.001, ****P < 0.0001, ANOVA with Tukey’s multiple comparison test.

A complication in studying PA-X N-terminal acetylation is that PA-X shares its N terminus with the PA subunit of the influenza RNA polymerase, a protein that is essential for viral replication (1, 2). This is because the ribosomal frameshifting event that generates PA-X occurs after translation of the 191st amino acid of PA, creating two proteins with the same N terminus and distinct C termini (1, 2). Therefore, mutations or acetylase knockdown/knockouts that alter PA-X N-terminal acetylation also impact PA N-terminal acetylation (10). Since PA also requires this modification for its function, due to an unknown mechanism of regulation, the original study by Oishi et al. was unable to separate the impact of PA-X vs*.* PA N-terminal acetylation during infection (10). Indeed, most of the experiments on PA-X activity in the Oishi et al. study were carried out with ectopically expressed PA-X (10). They did test the effect of NatB knockout on virus replication, but this knockout affects the acetylation of both PA-X and PA, as well as all other influenza and cellular proteins acetylated by NatB (10). Moreover, they did not test whether PA-X was still active during infection under these conditions (10).

Here, we report that N-terminal acetylation supports PA-X activity by two separate mechanisms. N-terminal acetylation is needed for PA-X nuclear localization (5, 8). This reveals a novel function for protein N-terminal acetylation. Moreover, we found that PA-X localizes to the nucleus when modified by Nat complexes that add the modification to either the initiator methionine or the second residue following initiator methionine excision. However, the localization alone is not enough to confer host shutoff activity, as PA-X mutants that are modified on the second residue are still inactive. PA-X thus specifically requires modification of the initiator methionine to downregulate RNA levels in cells. This result suggests that N-terminal acetylation on the initiator methionine has a separate role in supporting PA-X host shutoff activity. This is the first example of multiple roles of N-terminal acetylation for the same protein. Furthermore, we determined that PA-X activity also requires N-terminal acetylation in infected cells, pointing to a key role of this modification in immune regulation by influenza A virus.

## RESULTS

### N-terminal acetylation is required for PA-X nuclear localization

Previous studies using PA-X from the influenza A/WSN/33 H1N1 (WSN) virus have shown that N-terminal acetylation of PA-X by the NatB complex is important for its host shutoff activity in mammalian cells (10). When the N terminus of WSN PA-X is mutated from ME- to MP- to produce non-acetylated PA-X proteins (12, 14) (Fig. 1A), the shutoff activity of WSN PA-X is lost (10). One of the established functions of N-terminal acetylation is control of subcellular localization, particularly to the Golgi and the ER (35–38). Since we and others have reported that PA-X must localize to the nucleus to downregulate mRNAs (5, 8), we wondered whether N-terminal acetylation controlled PA-X localization and whether non-acetylated mutants lose activity because they accumulate in the cytoplasm. We thus compared the subcellular localization of ectopically expressed wild-type (WT) PA-X from the influenza A/Puerto Rico/8/1934 H1N1 virus (PR8) and the non-acetylated PR8 PA-X E2P mutant, both tagged at the C-terminus with eGFP, using confocal microscopy. We chose the PR8 variant because we have used it extensively for previous studies (5–7, 9), and it is 95% identical at the amino acid level to the WSN variant. We used mass spectrometry to confirm PA-X acetylation. For this and other mass spectrometry experiments, we used catalytically inactive PA-X D108A mutants to be able to express greater levels of protein and improve protein detection (expressing active PA-X at high levels is toxic to cells due to widespread RNA degradation). We observed that, as expected, the native E2-bearing N terminus is 100% acetylated, while the E2P mutant is not acetylated (Fig. 1B). To confirm that the PR8 PA-X E2P mutant loses the ability to reduce gene expression like the WSN PA-X E2P mutant (10), we co-transfected cells with PA-X and a luciferase reporter and measured luciferase RNA levels by qPCR, as we have done in previous studies (5, 6). As expected, the PA-X E2P mutant did not downregulate luciferase mRNA levels, similarly to the catalytically inactive PA-X D108A mutant (Fig. 1C) (5, 6). We then imaged the localization of eGFP-tagged WT PA-X, PA-X D108A, and PA-X E2P, as well as eGFP alone (Fig. 1D). To quantify PA-X localization, we computed the ratio of the mean fluorescence of eGFP in the nucleus vs. the cytoplasm from the confocal microscopy images using Image J/Fiji (39, 40). Ratios lower than 1 indicate primarily cytoplasmic localization, ratios close to 1 indicate diffuse cellular localization, and values greater than 1 indicate primarily nuclear localization (39). eGFP alone showed an average nuclear/cytoplasmic ratio of 1.28, indicating diffuse localization throughout cells, whereas WT PA-X was predominantly nuclear, with an average nuclear/cytoplasmic ratio of 2.92, consistent with previous studies and with the images (Fig. 1D and E) (5, 8). Interestingly, PA-X E2P was more diffusely localized than WT PA-X and had an average nuclear/cytoplasmic ratio of 1.42, similar to eGFP (Fig. 1D and E). These results suggest that N-terminal acetylation is required for the nuclear accumulation of PA-X, which could explain its loss of activity.

To ensure that the differences between WT PA-X and PA-X E2P were due to acetylation and not the sequence change, we also generated two mutants that have a different amino acid sequence but should still be modified by NatB, like WT PA-X: PA-X E2D and PA-X E2N (Fig. 1A). In both the WSN (10) and the PR8 (Fig. 1C) background, these NatB-modified mutants had similar host shutoff activity to WT PA-X, indicated by the ability to reduce expression of a co-expressed luciferase mRNA. Consistent with this result, PA-X E2D and PA-X E2N also had similar localization to WT PA-X, with nuclear/cytoplasmic ratios of 3.69 and 3.79, respectively (Fig. 1D and E). These results confirm that the differences seen between WT PA-X and PA-X E2P are due to changes in N-terminal acetylation and that N-terminal acetylation mediates nuclear localization of PA-X.

### Restoring nuclear localization of non-acetylated PA-X is not sufficient for host shutoff activity

Because nuclear localization is required for PA-X activity (5, 8), regulation of PA-X localization could explain why N-terminal acetylation is needed for PA-X function. To test this hypothesis and determine whether the decreased activity of PA-X E2P was solely due to its change in subcellular localization, we fused PA-X E2P to the canonical SV40 nuclear localization signal (41) to restore nuclear localization (Fig. 1D and E). PA-X E2P-NLS showed the expected nuclear localization (nuclear/cytoplasmic ratio = 5.56). However, it remained unable to downregulate luciferase mRNA levels (Fig. 1C). This result indicates that restoring the nuclear localization of an unmodified PA-X is not enough to restore its host shutoff activity. Therefore, we conclude that to downregulate RNA levels in cells, PA-X also requires N-terminal acetylation for a second function.

### N-terminal acetylation at any position is sufficient for PA-X localization to the nucleus, but not for host shutoff activity

To further explore the relationship between PA-X N-terminal acetylation, nuclear localization, and host shutoff activity, we mutated the second residue of PA-X to alter the Nat complex that modifies it (Fig. 1A). We chose to mutate PA-X rather than knock out the Nat complexes because 80-90% of the human proteome is N-terminally acetylated during translation, and knock-down of the complexes may have pleiotropic effects (13, 42, 43). We first tested the E2A, E2S, and E2V mutations, which lead to N-terminal acetylation by NatA (Fig. 1A). Unlike NatB-modified proteins, which are N-terminally acetylated at the initiator methionine, NatA-modified proteins are N-terminally acetylated at the second residue following methionine excision (44, 45) (Fig. 1A). A previous study showed that, like the predominantly non-acetylated WSN PA-X E2P mutant, the NatA-modified WSN PA-X E2A mutant loses its host shutoff activity (10), suggesting the initiator methionine needs to be retained for PA-X function. Using mass spectrometry, we confirmed acetylation of the E2A, E2S, and E2V mutants. PA-X E2A and E2S were 100% N-terminally acetylated at the second position following methionine excision, as expected (Fig. 2A). PA-X E2V showed a more varied pattern: the initiator methionine was only excised in about 80% of peptides (16 out of 21), and only about 90% (14 out of 16) of these were N-terminally acetylated. This is consistent with previous reports of partial N-terminal acetylation for proteins that start with MV- (42, 45). Like WSN PA-X E2A, PR8 PA-X E2A, E2S, and E2V had no apparent host shutoff activity in cells (Fig. 2B). Interestingly, despite losing the ability to downregulate mRNA levels, we saw that PA-X E2A, E2S, and E2V had a similar localization to WT PA-X and primarily accumulated in the nucleus (Fig. 2C and D). Taken together, these data suggest that N-terminal acetylation at any position and at high levels is sufficient for PA-X nuclear localization, but that the difference between modification by NatA vs*.* NatB is important for host shutoff activity.

### N-terminal acetylation at the initiator methionine by any Nat complex promotes PA-X host shutoff activity

**Fig 3:**
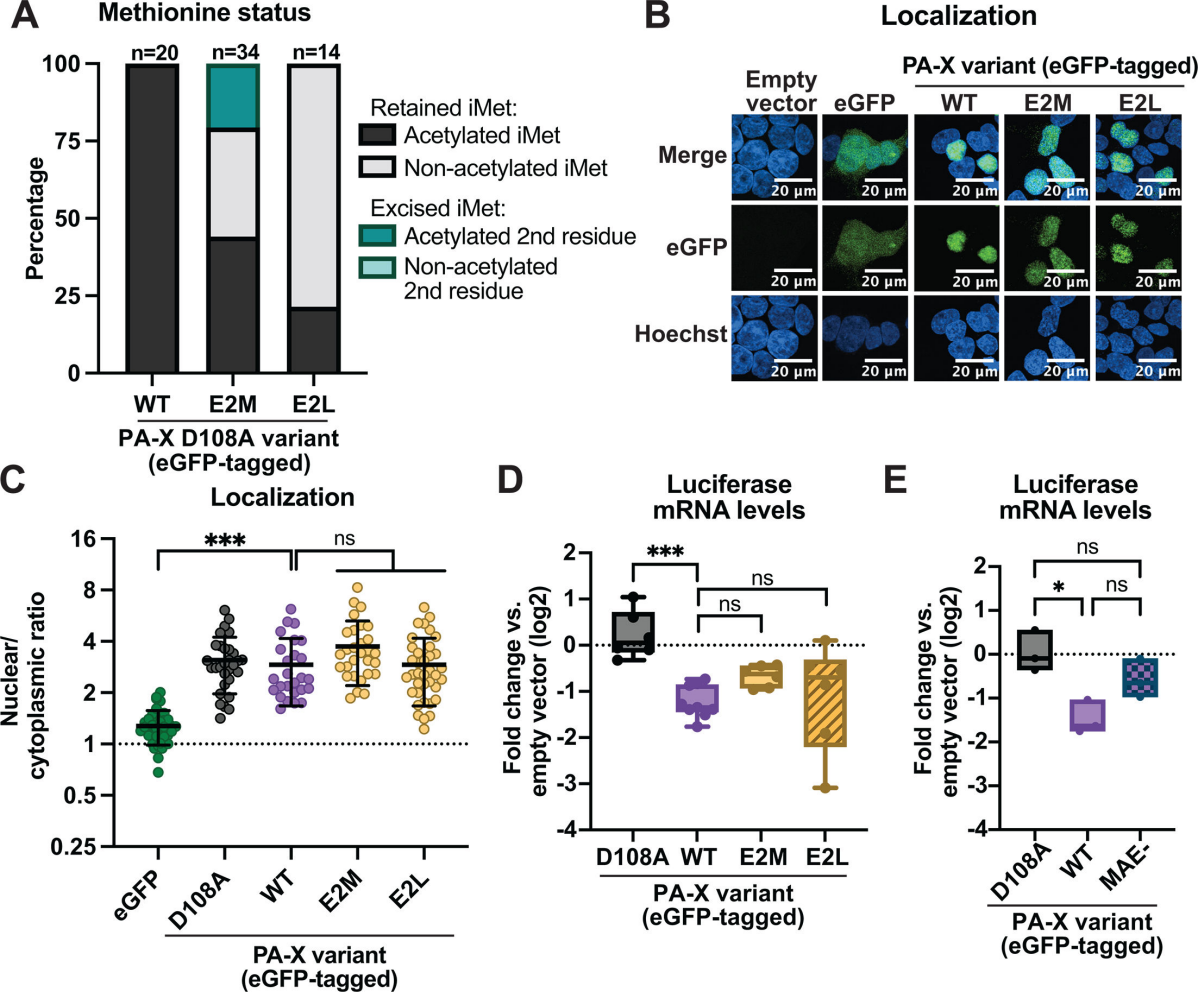
N-terminal acetylation at the initiator methionine promotes PA-X host shutoff activity. HEK293T cells were transfected for 24 h with empty vector, WT PR8 PA-X-eGFP, the catalytically inactive PA-X D108A-eGFP mutant, the indicated NatC-modified mutants carrying changes in the second amino acid in the WT or D108A PR8-PA-X-eGFP background, or the NatA-modified PR8 PA-X MAE-eGFP mutant, which has an alanine insertion between the native first and second amino acid. (**A**) eGFP-tagged proteins were isolated by immunoprecipitation, and peptides were analyzed by nanoLC-MS/MS for initiator methionine (iMet) retention and N-terminal acetylation. For these experiments, catalytically inactive versions of PA-X were used, and “WT” means WT sequence at aa 2. N above stacked bars indicates total detected peptides for each variant over two experiments. (**B**) Confocal microscopy was used to image the GFP signal in transfected cells. Nuclei were stained with Hoechst. (**C**) Nuclear/cytoplasmic ratios were calculated by measuring the mean fluorescence intensity of the nuclear and cytoplasmic compartments using ImageJ. Hoechst staining was used as a guide to generate regions of interest for the nucleus. Autofluorescence was used as a guide to generate cytoplasmic regions of interest. Nuclear/cytoplasmic ratios represent the following localization: <1: protein is primarily cytoplasmic, ~1: protein is diffuse throughout the cell, >1: protein is primarily nuclear. (**D and E**) mRNA levels of a co-transfected luciferase reporter were measured by RT-qPCR and normalized to 18S rRNA. Levels are plotted as fold change relative to vector-transfected cells in log2 scale. For all experiments, some conditions were carried out in parallel to the experiments in Fig. 1 and 2. Therefore, control conditions (empty vector, PA-X D108A, WT PA-X, and eGFP) for the RT-qPCR, mass spectrometry, and microscopy data and quantitation are identical, including representative images for empty vector, eGFP, and WT PA-X. *N* ≥ 3 for all experiments except the mass spectrometry analysis in panel A. For microscopy, representative images are shown. In nuclear/cytoplasmic ratio quantitation, each point represents 1–4 eGFP-positive cells, with 10–15 eGFP-positive cells quantified per condition in each biological replicate. ns = *P* > 0.05, *P < 0.05, ***P < 0.001, ANOVA with Tukey’s multiple comparison test.

It is surprising that PA-X mutants that are acetylated and localize to the nucleus (Fig. 2A, C and D) are inactive. Oishi et al. hypothesized that perhaps the NatB complex activity, rather than the modification, was important (10). However, NatA- and NatB-modified proteins also differ in the presence of the initiator methionine and thus the location of the modification (Fig. 1A). To test whether the site of the modification was the determining factor for PA-X activity, we investigated the effect of modification by NatC, which, like NatB, modifies proteins on the initiator methionine (13, 42). We generated two mutants that should be modified by NatC (12, 42), PA-X E2M and PA-X E2L (Fig. 1A). Mass spectrometry analysis showed that the initiator methionine of PA-X E2M was retained in 80% of peptides (27 out of 34), and about 60% of those peptides (15 out of 27) were N-terminally acetylated (Fig. 3A). For PA-X E2L, the initiator methionine was present on all detected peptides, but only 20% (3 out of 14) were N-terminally acetylated (Fig. 3A). The subcellular localization of the NatC-modified mutants was similar to that of WT PA-X (Fig. 3B and C). In addition, the NatC-modified mutants were able to downregulate luciferase mRNA levels in cells, although to a lesser degree than WT PA-X and the other NatB-modified mutants (Fig. 3D). The lower host shutoff activity may be linked to the incomplete acetylation of the proteins (Fig. 3A). Nonetheless, these results suggest that N-terminal acetylation of the initiator methionine specifically, and not NatB activity, is required for the host shutoff activity of PA-X.

While these results show that the difference between the NatA and NatB-modified proteins is not the complex that deposits the modification, there are still two differences between proteins modified by the two complexes—the removal of one amino acid in (M)A- vs. ME/D/N-starting proteins and the specific residue that is modified (M vs. A). Both features have the potential to change the surface of PA-X, which, in turn, may alter its interactions with RNA or proteins. To distinguish the effects of these two differences, we generated a mutant that should have its N-terminal methionine excised and start with an N-terminally acetylated alanine, while remaining the same length as WT PA-X. We achieved this by inserting an additional alanine between the initiator methionine and the second residue, generating the PA-X MAE- mutant. The PA-X MAE- mutant should be modified on the alanine by NatA after methionine excision. Thus, the only difference between WT PA-X and PA-X MAE- should be that the acetylation occurs on an alanine instead of a methionine. Interestingly, PA-X MAE- still largely lost the ability to downregulate luciferase mRNA levels (Fig. 3E). These results indicate that the initiator methionine itself is important.

### Neither eGFP tagging nor expression levels of the E2 mutant proteins explain the differences in localization or activity

**Fig 4:**
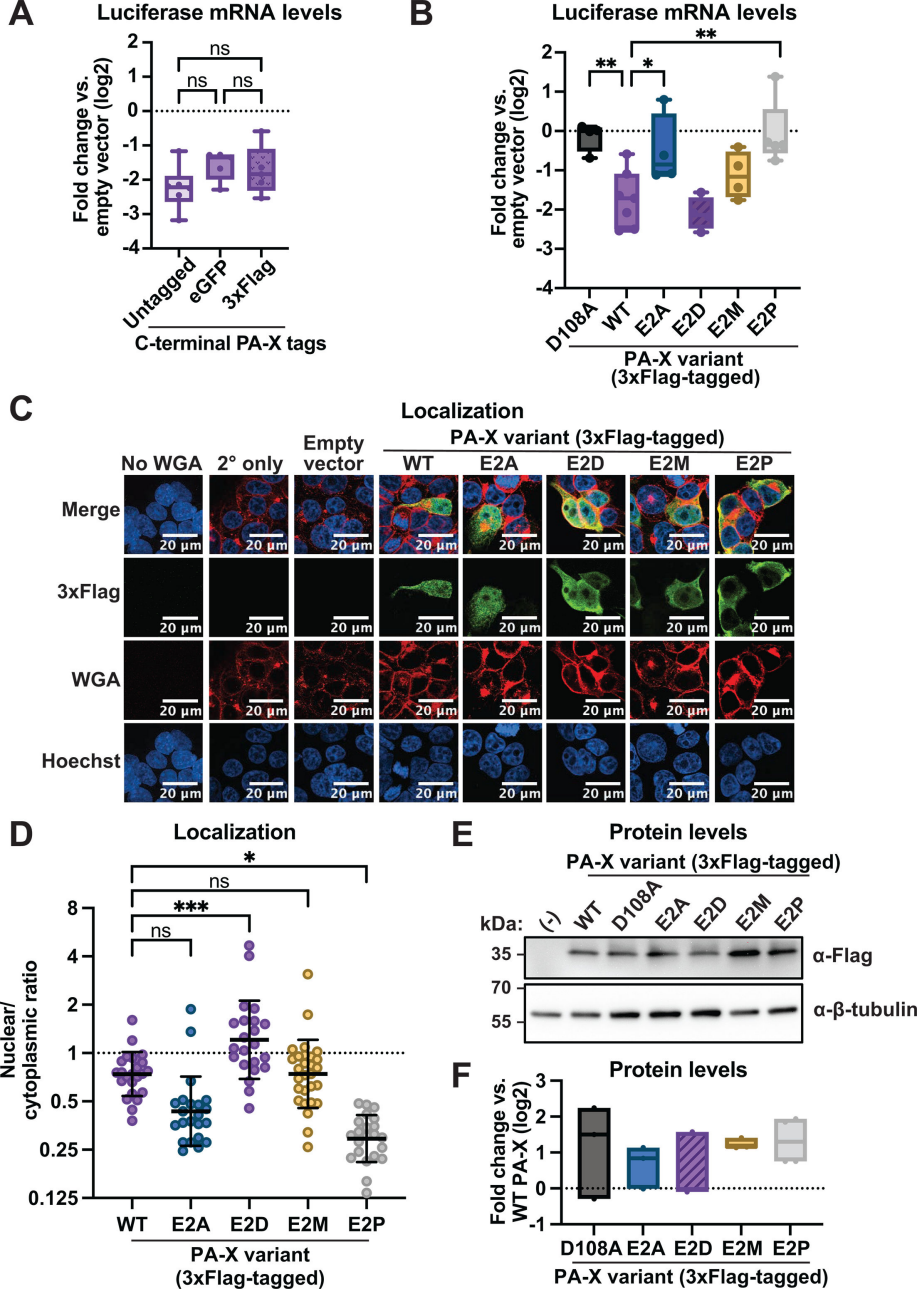
Differences in PA-X localization and activity are not due to the eGFP tag or variations in protein levels. HEK293T cells were transfected for 24 h with empty vector, untagged WT PR8 PA-X, PA-X-eGFP, PA-X-3xFlag, or the following 3xFlag-tagged PA-X mutants: E2A, E2D, E2M, or E2P. (**A and B**) mRNA levels of a co-transfected luciferase reporter were measured by RT-qPCR and normalized to 18S rRNA. Levels are plotted as fold change relative to vector-transfected cells in log2 scale. (**C**) Confocal microscopy was used to image transfected cells. Cell membranes were stained with WGA (red), PA-X was stained with α-Flag antibodies (green), and nuclei were stained with Hoechst (blue). (**D**) Nuclear/cytoplasmic ratios were calculated by measuring the mean fluorescence intensity of the nuclear and cytoplasmic compartments using ImageJ. Hoechst and WGA staining were used as guides to generate regions of interest for the nucleus and cytoplasm, respectively. (**E and F**) Protein levels were analyzed by Western blotting. A Flag antibody was used to visualize Flag-tagged PA-X and PA-X mutants. Tubulin staining was included as a loading control. (-) denotes empty vector transfection. An image representative of three replicates is shown in E. Images were quantified, and the average of three replicate experiments is shown in F. Levels are plotted as fold change relative to WT PA-X-3xFlag transfected cells in log2 scale. *N* ≥ 3 for all experiments. For microscopy and Western blots, representative images are shown. In nuclear/cytoplasmic ratio quantitation, each point represents 1–3 PA-X-positive cells, with 10–15 eGFP-positive cells quantified per condition in each biological replicate. ns = *P* > 0.05, *P < 0.05, **P < 0.01, ***P < 0.001, ANOVA with Tukey’s (**A**) or Dunnett’s (**B, D**) multiple comparison test.

Although the host shutoff activity of PA-X-eGFP was similar to that of untagged PA-X (Fig. 4A), we explored the possibility that our detection strategy for PA-X may lead to unexpected confounds due to the size of the eGFP tag. This is especially relevant for protein localization. We therefore tested whether a smaller C-terminal 3xFlag tag would yield the same results. Like PA-X-eGFP, WT PA-X-3xFlag had similar host shutoff activity to untagged PA-X (Fig. 4A). We generated PA-X E2A, PA-X E2P, PA-X E2D, and PA-X E2M 3xFlag-tagged proteins and found that they had similar host shutoff activity to their eGFP-tagged counterparts, corroborating our findings on the required role of N-terminal acetylation on the initiator methionine for activity (Fig. 4B). In immunofluorescence assays, WT PA-X-3xFlag and all the PA-X E2 mutant 3xFlag-tagged proteins had a more diffuse localization than their eGFP-tagged counterparts (Fig. 4C). While this could be due to the tag difference, we note that for the immunofluorescence assay, we transfected cells with 16X the amount of PA-X-3xFlag containing plasmids to ensure detection. This likely increases the overall levels of PA-X and thus the portion of PA-X found outside the nucleus. Nonetheless, the E2 mutations altered Flag-tagged PA-X localization in an analogous manner to that of eGFP-tagged PA-X. WT PA-X, PA-X E2D (both NatB-modified), and PA-X E2M (NatC-modified) had similar nuclear/cytoplasmic ratios, approximately 0.8, 1.4, and 0.8, respectively. PA-X E2A (NatA-modified) was slightly less nuclear (ratio ~0.5), although this difference did not reach statistical significance when compared to WT PA-X (Fig. 4D). In contrast, the unmodified PA-X E2P-3xFlag was significantly less nuclear than WT PA-X (ratio ~0.3, Fig. 4D). Overall, the similar trends between the eGFP-tagged and 3xFlag-tagged PA-X E2 mutants support the conclusion that N-terminal acetylation at any position is sufficient for PA-X nuclear localization.

Although we were able to detect all the eGFP-tagged PA-X variants by microscopy, which suggested that they were expressed at similar levels, we have struggled to detect them consistently by Western blotting. Therefore, we have previously been unable to verify that they are expressed at similar levels and rule out that N-terminal acetylation affected PA-X stability. We tested whether the 3xFlag tag**,** which has been used successfully by others (8), gave us better detection. Indeed, we found that we could easily visualize Flag-tagged PA-X on a Western blot. Moreover, we were able to confirm that WT and the E2 mutants were expressed at similar levels, including the inactive E2A and E2P mutants (Fig. 4E and F). Therefore, we can conclude that protein expression or stability differences do not explain activity or localization changes due to these mutations.

### N-terminal acetylation by different Nat complexes has similar consequences on the activity of PA-X of all influenza A strains tested

**Fig 5:**
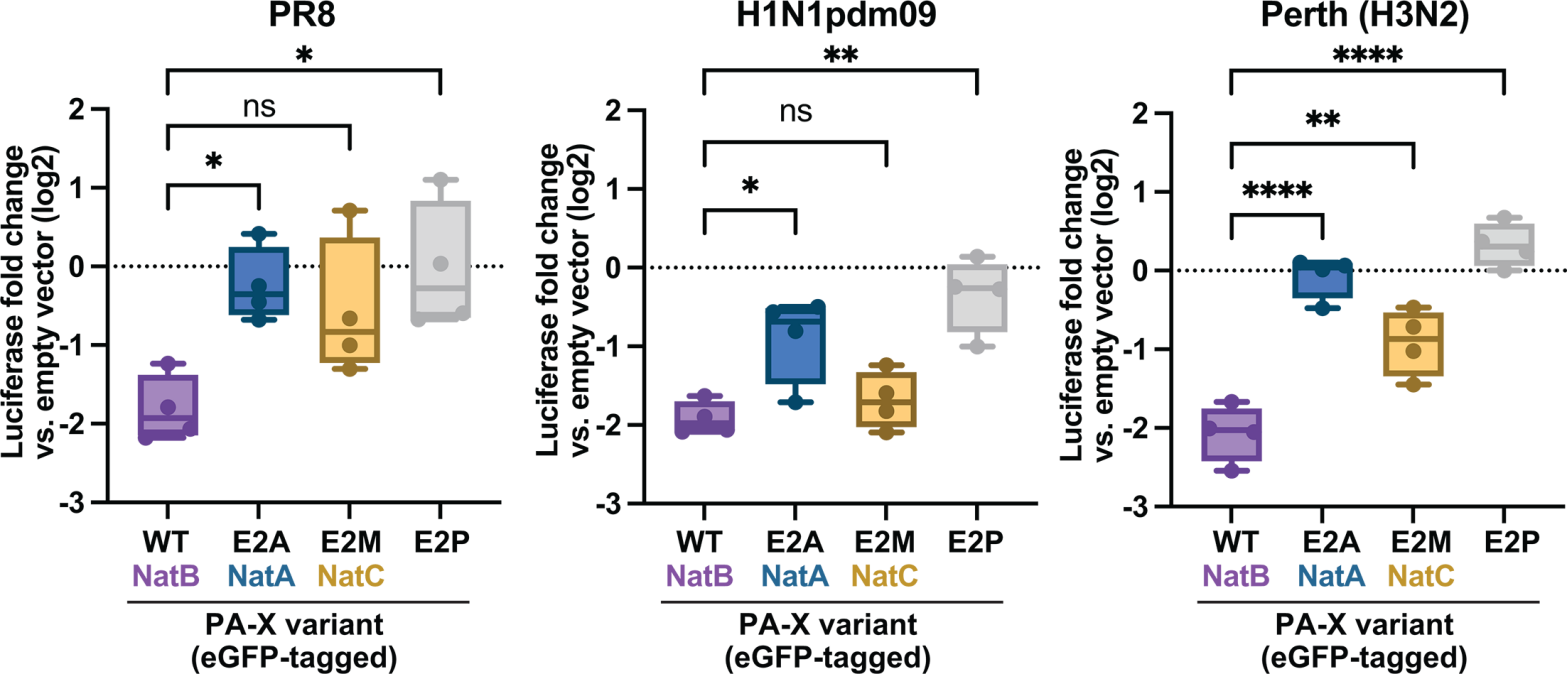
N-terminal acetylation also regulates the activity of PA-X proteins from circulating influenza A virus subtypes. HEK293T cells were transfected for 24 h with empty vector, WT PR8 PA-X-eGFP, WT H1N1pdm09 PA-X-eGFP, or WT Perth PA-X-eGFP or the corresponding PA-X-eGFP mutants carrying changes in the second amino acid. mRNA levels of a co-transfected luciferase reporter were measured by RT-qPCR and normalized to 18S rRNA. Levels are plotted as fold change relative to vector-transfected cells in log2 scale. *n* = 4. ns = *P* > 0.05, *P < 0.05, **P < 0.01, ****P < 0.0001, ANOVA with Tukey’s multiple comparison test.

While results with WSN (10) and PR8 PA-X are suggestive of a broader phenomenon, both of these strains are lab-adapted viruses. Therefore, we sought to ensure that the phenotype of N-terminal acetylation mutants was conserved in PA-X proteins from more relevant human seasonal influenza viruses. Currently circulating seasonal human viruses are H1N1 strains related to the pandemic 2009 H1N1 virus (H1N1pdm09) and H3N2 strains. Thus, we tested the effect of E2P (unmodified), E2A (NatA-modified), and E2M (NatC-modified) mutations in the PA-X sequences from A/Tennessee/1-560/2009 H1N1 (H1N1pdm09) and A/Perth/16/2009 H3N2 (Perth). We found that changes in N-terminal acetylation patterns had a similar effect on PA-X proteins from all influenza A strains tested. All PA-X E2A and PA-X E2P mutants had significantly lower (or no) host shutoff activity, while the PA-X E2M mutants maintained an intermediate amount of activity (Fig. 5). These results suggest that, regardless of viral strain, PA-X requires N-terminal acetylation in general to access the nucleus and N-terminal acetylation of the initiator methionine specifically to downregulate RNA levels.

### N-terminal acetylation is required for PA-X host shutoff activity during infection

**Fig 6:**
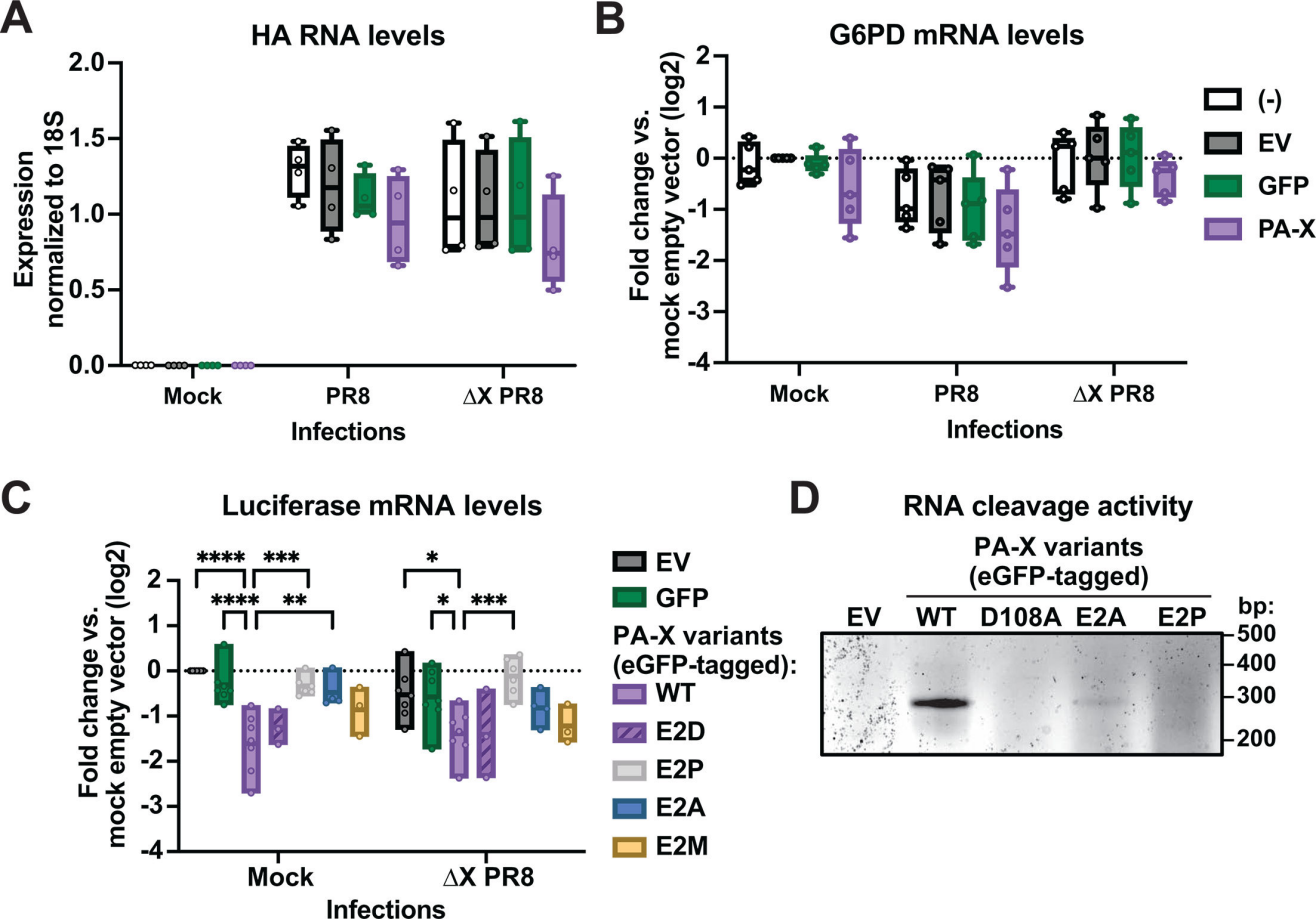
N-terminal acetylation of PA-X is required for host shutoff activity during infection. (**A–C**) HEK293T cells were left untrasfected [(-)] or transfected for 24 h with empty vector (EV), eGFP, and eGFP-tagged WT PA-X or the following eGFP-tagged PA-X mutants: unmodified E2P mutant (gray), NatB-modified E2D mutant (purple), NatA-modified E2A mutant (blue), or NatC-modified E2M mutant (yellow). 8 h after transfection, cells were mock infected or infected with WT PR8 or PA-X-deficient PR8 (∆X) at an MOI of 1. RNA samples were collected 16 h post-infection (24 h post-transfection). RNA levels of (**A**) viral HA RNA, (**B**) endogenous G6PD mRNA, and (**C**) the co-transfected luciferase reporter were measured by RT-qPCR and normalized to 18S rRNA. For B and C, levels are plotted as a change relative to vector-transfected cells in a log2 scale. *n* ≥ 3. In A and B, none of the comparisons were statistically significant. *P < 0.05, **P < 0.01, ***P < 0.001, ****P < 0.0001, Ordinary two-way ANOVA with Šidák’s multiple comparison test compared to the WT PA-X in each infection condition. (**D**) HEK293T ishXRN1 cells were transfected for 24 h with empty vector and the following eGFP-tagged constructs: WT PA-X, PA-X D108A (catalytically inactive), PA-X E2A, or PA-X E2P, alongside the pCMV-luciferase + intron-YKT6-99bp cleavage reporter. RNA samples were collected 24 h post-transfection, and 5′ RACE was performed. Bands seen on the agarose gel correspond to the predicted size of the PCR product corresponding to the cleaved fragment (290 bp), and its provenance was verified by Sanger sequencing. The agarose gel image is representative of three biological experiments.

Our results point to the importance of PA-X N-terminal acetylation for nuclear localization and host shutoff activity when PA-X is ectopically expressed in cells. However, we wanted to confirm that this modification is also important for host shutoff during viral infection. This was not done in the previous study examining the WSN variant by Oishi et al. (10) because of the challenges in separating PA and PA-X modifications during infection. PA-X is produced by a +1 frameshifting event that occurs after translation of the 191st amino acid of PA (1, 2). Therefore, PA and PA-X have the same N terminus, and any mutation in the PA-X N terminus is also present in PA (1, 2). This is problematic as PA also requires N-terminal acetylation for polymerase activity (10). Similarly, both proteins are affected by the knockout of the NatB complex, which was used by Oishi et al. to measure the impact of N-terminal acetylation on viral kinetics (10). Thus, to separate PA and PA-X production, we examined the activity of ectopically expressed PA-X mutants in cells infected with PA-X-deficient PR8 (PR8 PA(∆X)) (6). While the human lung epithelial cell line A549 is most commonly used as a model for influenza infection, transfection efficiency is low in these cells. Therefore, we used HEK293T cells, which are readily transfectable and have been used in many previous studies of influenza A virus infections (46–51). We have also observed that PR8 infections are more efficient in HEK293T than A549 cells (40% or more cells expressing the viral nucleoprotein 20 h post-infection vs. less than 5%), which facilitates downstream analysis of cell-intrinsic reduction in RNA levels. To ensure that the experimental setup would work, we first checked for potential interference between transfection and infection. We transfected HEK293T cells with plasmids encoding eGFP or eGFP-tagged WT PA-X, left them uninfected or infected them with WT PR8 or PR8 PA(∆X), and tested for the efficiency of PA-X host shutoff and of infection. We found that transfecting cells did not prevent them from being infected with WT PR8 or PR8 PA(∆X) viruses, as there were no statistically significant differences in the levels of influenza HA RNAs (Fig. 6A). Moreover, transfection did not inhibit the ability of virus-encoded PA-X to downregulate endogenous targets. G6PD, a known target of PA-X (6), was downregulated in transfected cells that were infected with WT PR8, but not PR8 PA(∆X) (Fig. 6B). We note that, as we have found throughout our work, the <100% rate of infection in the cell population means that G6PD downregulation is underestimated due to the presence of many cells that are not infected and have normal G6PD levels. Having established these initial parameters, we repeated the experiment and transfected cells with a luciferase reporter and eGFP-tagged versions of WT PA-X, PA-X E2D, which is still modified by NatB, PA-X E2P, which is unmodified, or eGFP alone. Cells were then infected with PR8 PA(∆X) virus or mock infected (Fig. 6C). To examine PA-X activity, we used a transfected luciferase reporter rather than an endogenous gene, because the reporter allows us to measure RNA downregulation specifically in cells that express the co-transfected PA-X. This reduces confounds from untransfected cells, where there is no RNA downregulation. As expected, WT PA-X was active and downregulated luciferase mRNA levels in both mock and PR8 PA(∆X)-infected cells, and the E2D mutant had similar activity as WT PA-X under both conditions. In contrast, based on luciferase mRNA downregulation, unmodified PA-X E2P was inactive in both mock and PR8 PA(∆X)-infected cells. This result shows for the first time that PA-X requires N-terminal acetylation for activity during infection.

We also tested the PA-X E2A (NatA-modified) and E2M (NatC-modified) mutants and found that their host shutoff activity during infection recapitulated the patterns seen in ectopic expression. In both conditions, the RNA downregulation activity of E2M mutants was similar to that of WT PA-X, whereas E2A mutants had reduced activity (Fig. 6C). That said, during infection with PR8 PA(∆X), luciferase downregulation by PA-X E2A was not significantly different from downregulation by WT PA-X, whereas there was still a significant difference between WT PA-X and PA-X E2P. Therefore, it is possible that in infected cells, there is potentiation of PA-X activity that is revealed by examining less active mutants or variants. In turn, this suggests that PA-X E2A retains some ability to degrade RNA, whereas PA-X E2P is completely inactive.

To test the RNA cleavage activity of the PA-X E2A and E2P mutants, we mapped fragments of PA-X degradation using 5′ rapid amplification of DNA ends (5′ RACE), as we did previously to study PA-X targeting preferences (7). We analyzed cleavage of the PA-X target sequence of the human YKT6 mRNA within a luciferase reporter, as we have extensively characterized PA-X cleavage specificity using this sequence and this reporter (7). Ectopically expressed PA-X E2A was able to cleave mRNA targets at the previously identified PA-X target site, whereas PA-X E2P did not have apparent RNA cleavage activity (Fig. 6D), even though neither downregulated mRNA levels by RT-qPCR (Fig. 1C, 2B and 6C). In two out of three biological replicates, including the one shown in Fig. 6D, cleavage by the E2A mutant appeared reduced compared to cleavage by WT PA-X. However, 5′ RACE is an endpoint PCR-based assay and does not accurately reveal quantitative differences, providing only a binary cleavage or no cleavage readout. Therefore, we cannot confidently conclude that the E2A mutation reduces RNase activity. Nonetheless, this result suggests that PA-X E2A mutants still have some RNase activity but somehow cannot properly reach their targets in uninfected cells. This is consistent with the observation that PA-X E2A localizes to the correct compartment, whereas PA-X E2P does not (Fig. 1 and 2), and with previous results indicating that PA-X predominantly degrades its target RNAs in the nucleus (5, 8). Moreover, it further underscores the difference between N-terminal acetylation at the initiator methionine, N-terminal acetylation after methionine excision, and no N-terminal acetylation, and the complex role of this modification in modulating PA-X activity.

## DISCUSSION

Our results reveal that N-terminal acetylation of PA-X supports host shutoff activity in two separate ways: it is required for nuclear localization of PA-X, and it is involved in a second yet undetermined process that promotes PA-X activity. Interestingly, the two processes have slightly different requirements for the modification. The addition of the acetyl group at the first residue, the initiator methionine, or the second residue following initiator methionine excision both promote nuclear localization of PA-X (Fig. 1D and E, 2C and D). However, mutants that are modified at the second residue, like PA-X E2A, still have reduced host shutoff activity and do not strongly downregulate RNA levels (Fig. 2B), despite being localized to the nucleus (Fig. 2C and D) and retaining RNase activity (Fig. 6D). Further mutational analysis indicates that the methionine residue at the beginning of the protein is important, rather than the change in protein size and potentially protein surface (Fig. 3E). Thus, we demonstrate that an acetyl group on the initiator methionine is specifically required for the full host shutoff activity of PA-X. In addition, we were able for the first time to directly test the importance of N-terminal acetylation for PA-X activity in infected cells, and to demonstrate that these findings are relevant to influenza A virus infection.

The previous study on PA-X N-terminal acetylation had concluded that NatB activity was specifically important for PA-X function, as they also observed that the E2A substitution abolished PA-X host shutoff activity of PA-X despite the N-terminal acetylation of PA-X E2A (10). Our mutant analysis shows that this interpretation is likely not correct. Instead, our results suggest that the acetylated methionine is structurally different from an acetylated alanine and that the former is needed for PA-X function. To our knowledge, this is the first study to suggest that equivalent levels of acetylation by different Nat complexes are not interchangeable. The difference between WT PA-X and PA-X E2A is likely linked to changes to protein-protein interactions (31–34). This conclusion is supported by the observation that changes in N-terminal acetylation do not reduce PA-X protein accumulation (Fig. 4E and F), even though this is an important function of this modification (17–25). Biochemically, N-terminal acetylation eliminates the N-terminal positive charge of the first amino acid and increases hydrophobicity. This may allow for a new protein interaction surface that is unavailable when the N-terminus is unmodified. This would be analogous to the function of N-terminal acetylation of the NEDD8-conjugating E2 enzyme Ubc12, which allows for its docking into the hydrophobic pocket of the co-E3 ligase Dcn1 (32, 33). The structure of the interaction interfaces may also be subtly different depending on whether the initiator methionine is retained.

Since PA-X E2A retained the correct localization despite losing activity, our study also reveals that N-terminal acetylation is required for multiple separable aspects of PA-X biology, another feature that departs from previous reports of N-terminal acetylation. How this occurs remains unclear. Changes in protein-protein interactions are also likely to underlie these requirements. PA-X may interact with different proteins for its nuclear import *vs*. its association with targets. N-terminal acetylation at the first or second amino acid may support interactions with a cytoplasmic protein that is involved in the nuclear import of PA-X. However, retention of a modified methionine may be needed to create the correct interaction surface for a protein that allows PA-X to reach, bind, and/or degrade its RNA targets in the nucleus. These possibilities should be addressed in future studies.

To note, this is the first time that a role for the N-terminal region of PA-X in nuclear localization has been reported. Previous studies have identified charged residues in the C-terminal X-ORF of PA-X that are important for nuclear localization (5, 8, 52). Here, we show that these sequences alone are not enough for PA-X nuclear localization, as they were not mutated in our studies. Conversely, N-terminal acetylation is also not enough to direct PA-X nuclear localization, because the N-terminal domain of PA-X alone does not traffic to the nucleus (5, 8), even though it is likely still N-terminally acetylated by NatB. The full structure of PA-X with its C-terminal X-ORF has not yet been solved, and further work should determine how the acetylated N-terminus and the C-terminal X-ORF work together to drive the nuclear localization of PA-X.

Our study is also the first report of the regulation of nuclear localization by N-terminal acetylation. Other post-translational modifications like phosphorylation (53, 54), arginine methylation (55–57), and lysine acetylation (58) are well known to modulate nuclear localization, largely by mediating interactions with importin α, which mediates nuclear import of proteins. It is possible that the N-terminal acetylation similarly increases binding affinity between PA-X and interacting proteins that mediate nuclear localization. N-terminal acetylation has been described to regulate protein localization to membranes, through protein-protein interactions with integral membrane proteins (36, 38) or direct interactions with membranes (59). These results thus suggest that N-terminal acetylation has a broader range of functions in protein localization than previously reported and mediates interactions with additional trafficking proteins.

Using PA-X rescue in *trans,* we were able to separate the role of PA vs. PA-X N-terminal acetylation during infection and demonstrate that PA-X requires N-terminal acetylation at the initiator methionine for normal host shutoff during infection. Importantly, the glutamic acid at the second position of PA-X is highly conserved among influenza isolates, with 99.7% of H1N1pdm09 viruses, 99.9% of H3N2 viruses, 100% of H5N1, and 100% of H7N9 viruses containing this amino acid at the second position (10). As proteins starting in ME- are efficiently N-terminally acetylated (Fig. 1B), it is likely that all variants of PA-X carry this modification. The high conservation underscores how efficient N-terminal acetylation of PA-X is important for the host shutoff activity of PA-X, as not all other sequences are efficiently acetylated (Fig. 2A and 3A). Our investigation of PA-X from multiple strains also supports a role for N-terminal acetylation across a range of influenza A strains. Interestingly, during infection, the activity of the NatA-modified PA-X E2A mutant was not statistically different from WT PA-X activity. PA-X E2A, which retains at least some RNA cleavage activity (Fig. 6D), may be somewhat more active during infection. Therefore, this mutant could be used in the future to reveal additional factors during infection that support PA-X activity.

Overall, we have discovered that N-terminal acetylation functionally promotes the host shutoff activity of PA-X by separately influencing PA-X subcellular localization and its overall ability to downregulate target mRNAs. Our results also indicate that acetylation of the initiator methionine specifically promotes RNA downregulation by PA-X. In the future, it will be important to determine how N-terminal acetylation contributes to structural differences and/or PA-X interactions with other proteins and PA-X substrates. Additionally, in the context of cellular biology, these studies contribute new evidence that N-terminal acetylation supports the correct subcellular localization of proteins and, to our knowledge, constitute the first report that N-terminal acetylation promotes nuclear localization. Our studies also highlight how N-terminal acetylation is multifaceted in its regulation of modified proteins and how this modification remains incompletely understood, limiting our ability to discern its role in the regulation of proteins in physiological and pathological settings.

## MATERIALS AND METHODS

### Plasmids

C-terminally tagged pCR3.1-PA-X-eGFP and pCR3.1-PA-X D108A-eGFP from the PR8 strain were gifts from C. McCormick (Dalhousie University, Halifax, NS, Canada) and generated as previously described (60). pCDNA3.1-eGFP and pCDNA4/TO-C-terminal 3xFlag were a gift from B. Glaunsinger (University of California, Berkeley, Berkeley, CA, USA). The luciferase construct with the β-globin intron (pCMV luciferase + intron) was a gift from G. Dreyfuss (University of Pennsylvania, Philadelphia, PA, USA) (61). The pCMV-luciferase + intron-YKT6-99bp construct containing a strong PA-X cut site from the human YKT6 gene within the luciferase mRNA was previously described (7). The rescue plasmids encoding the eight segments of PR8 virus (pHW191-PB2 to pHW198-NS) were gifts from R. Webby (St. Jude Children’s Research Hospital, Memphis, TN, USA) (62). Gibson assembly using the HiFi assembly mix (New England Biolabs) was used to make pCR3.1-PA-X-NLS-eGFP by amplifying the PR8 sequence of PA-X from pCR3.1-PA-X-myc and the eGFP sequence from pCDNA3.1-eGFP. The NLS in pCR3.1-PA-X-NLS-eGFP was designed into the primers for Gibson assembly. From this construct, we then generated the PA-X E2P-NLS mutant using QuikChange II site-directed mutagenesis (Agilent). eGFP fusion constructs for PA-X from non-PR8 strains were made by replacing the PR8 PA-X sequence in pCR3.1-PA-X-eGFP with the PA-Xs from A/Perth/16/2009 H3N2 (Perth) and A/Tennessee/1-560/09 H1N1 (H1N1pdm09) subcloned from previously published myc-tagged constructs (5, 7) using the MluI/SalI restriction sites and T4 DNA ligation (New England Biolabs). The E2 mutations and the MAA- and MAE- insertions in the pCR3.1-PA-X-eGFP constructs with PR8 PA-X, PR8 PA-X D108A, Perth PA-X, and H1N1pdm09 PA-X were generated using QuikChange II site-directed mutagenesis (Agilent). The C-terminal 3xFlag-tagged PA-X constructs were generated by amplifying WT and E2 mutant PR8 PA-X sequences from the pCR3.1 backbones and inserting them into the HindIII and NotI sites of pCDNA4/TO-C terminal 3xFlag vector using Gibson cloning (HiFi assembly mix, New England Biolabs). The PR8 pHW-PA(∆X) plasmid, previously described (6, 7), was generated from pHW193 by introducing mutations that reduce frameshifting events and add a premature stop codon in the PA-X reading frame, but that are silent in the PA reading frame.

### Cell lines and transfections

HEK293T cells and Madin-Darby canine kidney (MDCK) cells were commercially obtained from ATCC (CRL-3216 and CCL-34, respectively). HEK293T cells expressing inducible short hairpin RNA against XRN1 (ishXRN1) were previously described (63). All cell lines were maintained in high-glucose Dulbecco’s modified Eagle’s medium (DMEM, Gibco) supplemented with 10% fetal bovine serum (FBS, Cytiva) at 37°C in 5% CO2 atmosphere. To measure host shutoff activity during transfection, HEK293T cells were plated on 24-well plates and transfected with 500 ng total DNA (including 25 ng PA-X constructs and 50 ng pCMV luciferase + intron construct) in 10% FBS in DMEM using jetPRIME transfection reagent (Polyplus). Cells were collected 24 h post-transfection for RNA extraction and purification. To determine the subcellular localization of PA-X using eGFP-tagged proteins, HEK293T cells were transfected as described above. To determine the subcellular localization of PA-X using Flag-tagged proteins, HEK293T cells in 12-well plates were transfected with 500 ng of PA-X constructs. In both cases, they were plated onto poly-L-lysine (Sigma)-treated glass coverslips. To measure direct cleavage of RNAs by RACE, HEK293T ishXRN1 cells were treated with 1 μg/mL doxycycline for 3–4 days to induce the XRN1 shRNA and knock down the protein. They were then plated in six-well plates and transfected with 2 μg total DNA, including 125 ng of PA-X constructs and 250 ng of the pCMV-luciferase+intron-YKT6-99bp cleavage reporter, similarly to what was done in Gaucherand et al. (7). To measure the host shutoff activity of PA-X during influenza A virus infections, HEK293Ts were plated in 24-well plates that were pre-treated with fibronectin, bovine serum albumin (BSA), and bovine collagen to increase HEK293T adhesion to the plate during the procedure. For the plate treatment, 10 μg/mL fibronectin (Sigma-Aldrich), 100 μg/mL BSA (Sigma-Aldrich), and 30 μg/mL bovine collagen (Advanced Biomatrix) in modified Eagle’s medium (MEM, Gibco) were added to the wells and UV crosslinked for 30 min. Wells were then washed three times with Dulbecco’s phosphate-buffered saline (DPBS, Gibco) before the addition of the cells. After plating, cells were transfected with 500 ng total DNA (including 25 ng PA-X constructs and 50 ng pCMV luciferase + intron construct) in infection media (0.5% low-endotoxin BSA [Sigma-Aldrich] in high glucose DMEM) using jetPRIME transfection reagent. After 8 h of transfection, the media was removed, and the cells were infected as described below. After 24 h of transfection/16 h post-infection, cells were collected for RNA extraction and purification.

### Viruses and infections

WT influenza A virus A/Puerto Rico/9/1934 H1N1 (PR8) and the mutant recombinant virus PR8 PA(∆X) were generated using the eight-plasmid reverse genetic system (64) as previously described (5–7). Viral stocks were produced in MDCK cells, and infectious titers were determined by plaque assays in MDCK cells using 1.2% Avicel (FMC BioPolymer) overlays (65). Briefly, confluent MDCK cells were infected with low volumes of 10-fold serially diluted virus stocks in triplicate for 1 h. Cells were then washed twice with DPBS before the addition of overlay media (1.2% Avicel, 1× MEM, 0.5% low-endotoxin BSA (Sigma-Aldrich), and 1 μg/mL TPCK-treated trypsin (Sigma-Aldrich)) and incubated for 4 days at 37°C in 5% CO_2_ atmosphere. After 4 days, cells were fixed with 4% paraformaldehyde and stained with crystal violet (Sigma-Aldrich) to observe plaques. Influenza A virus infections following HEK293T transfections were performed in infection media (0.5% low-endotoxin BSA in high glucose DMEM). Briefly, transfected HEK293T cells were treated with infection media supplemented with 0.5 μg/mL TPCK-treated trypsin alone (mock infection) or containing WT PR8 or PR8 PA(∆X) at a multiplicity of infection (MOI) of 1 and incubated for 16 h at 37°C in 5% CO_2_ atmosphere. Cells were then collected and lysed in RNA lysis buffer for RNA isolation.

### RNA purification, cDNA preparation, and qPCR

RNA was extracted and purified using the Quick-RNA miniprep kit (Zymo Research) following the manufacturer’s protocol. Purified RNA was treated with Turbo DNase (Life Technologies), then reverse transcribed using iScript supermix (Bio-Rad) per the manufacturer’s protocol. qPCR was performed using iTaq Universal SYBR Green Supermix (Bio-Rad) on the Bio-Rad CFX Connect Real-Time PCR Detection System or CFX Duet Real-Time PCR System and analyzed with Bio-Rad CFX Manager software or Bio-Rad CFX Maestro software. 18S ribosomal RNA was chosen as a housekeeping gene for normalizing data because its levels are stable during influenza A virus infections (66) and because rRNAs are not targeted by degradation by PA-X (5). Primers used are listed in Table 1.

**TABLE 1:** qPCR primers used in this study

Primer name	Primer sequence (5′→3′)	Reference
18S forward	GTAACCCGTTGAACCCCATT	Abernathy et al. (67)
18S reverse	CCATCCAATCGGTAGTAGCG	Abernathy et al. (67)
Luciferase forward	ATCGAGGTGGACATTACCTACG	Khaperskyy et al. (5)
Luciferase reverse	CGCTCGTTGTAGATGTCGTTAG	Khaperskyy et al. (5)
Human G6PD forward	TGAGCCAGATAGGCTGGAA	Hu et al. (68)
Human G6PD reverse	TAACGCAGGCGATGTTGTC	Hu et al. (68)
PR8 HA forward	CTGGACCTTGCTAAAACCCG	Slaine et al. (69)
PR8 HA reverse	TCTGGAAAGGGAGACTGCTG	Slaine et al. (69)

### Immunofluorescence assays and confocal microscopy

HEK293T cells were grown on glass coverslips pretreated with poly-L-lysine (Sigma-Aldrich), which were prepared following the manufacturer’s protocol. After 24 h of plating, cells were transfected with PA-X variants tagged at the C terminus with eGFP or a 3xFlag tag following the protocols described above. After 24 h of transfection, cells were washed twice with PBS and fixed with 4% paraformaldehyde. For PA-X-eGFP analysis, cells were then permeabilized in 0.1% Triton X-100, and nuclei were stained with 1:10,000 dilution of Hoechst 3342 Fluorescent Stain (Fisher Scientific) for 10 min at room temperature. For PA-X-3xFlag staining, after fixation, the cell membrane was stained by incubating cells for 10 min in 2.5 μg/mL wheat germ agglutinin (WGA) conjugated to Alexa Fluor 647 (Fisher Scientific, W32466) in PBS at room temperature in the dark. They were then permeabilized with 0.1% Triton X-100 in PBS for 10 min, blocked with 10% BSA in PBS for 30 min at 37°C, and incubated with 1:500 anti-Flag antibody (M2 clone, Sigma Aldrich F1804) in 3% BSA in PBS for 2 h at room temperature. Stained cells were washed in PBS and incubated with 5 μg/mL goat anti-mouse secondary antibody conjugated to Alexa Fluor 488 (ThermoFisher, A11029) in 3% BSA in PBS for 45 min at room temperature. Nuclei were stained with a 1:10,000 dilution of Hoechst 3342 Fluorescent Stain (Fisher Scientific) for 10 min at room temperature. For all experiments, coverslips were then mounted on glass slides using ProLong Gold Antifade Mountant (Thermo Fisher). Images were taken with the Nikon A1R Confocal Microscope or the Leica Stellaris Confocal Microscope. To analyze PA-X subcellular localization, Image J/Fiji (39, 40) was used to draw regions of interest for the nuclear and cytoplasmic compartments for individual cells, or if not possible, groups of up to four cells. All GFP/Flag-positive cells in an image were analyzed. Hoechst and autofluorescence (for PA-X-GFP images) or WGA staining (for PA-X-3xFlag images) were used to define nuclear and cytoplasmic compartments, respectively. The mean fluorescence intensity of eGFP or Flag signal in each compartment was then measured, and the nuclear/cytoplasmic ratio of the mean intensity measurements was calculated. 10–15 cells per condition were analyzed for each of at least three replicate experiments. Individual cell measurements rather than experimental averages were used for plotting and statistical analysis.

### Protein collection and Western blotting

Cell lysates were prepared using RIPA buffer (50 mM Tris-HCl pH 7.4, 1% NP-40 alternative, 0.5% sodium deoxycholate, 0.1% SDS, 150 mM NaCl, 2 mM EDTA) supplemented with 1× cOmplete EDTA-free protease inhibitor cocktail (Roche), 2 mM MgCl_2_, and 250 U/mL benzonase nuclease (Sigma-Aldrich). 100 μg of protein was loaded on Mini-PROTEAN TGX Precast Protein Gels, 4%–15% (10-well) or Mini-PROTEAN TGX Precast Protein Gels, 4%–20% (15-well) SDS-PAGE gels (Bio-Rad) and transferred onto polyvinylidene difluoride membranes (Millipore) using a Trans-Blot Turbo Transfer System (BioRad). Membranes were blocked with 5% milk in Tris buffer saline (TBS) with 0.1% Tween-20 (TBST). Western blots were performed at 4°C overnight using antibodies against Flag tag (Sigma-Aldrich F1804, 1:500) and β-tubulin (Cell Signaling Technologies #2128, 1:1000) diluted in 0.5% milk in TBST. Secondary antibodies conjugated to horseradish peroxidase (SouthernBiotech) were used at a 1:5,000 dilution, and a chemiluminescent signal was generated using the Pierce ECL Western Blotting Substrate (Thermo Fisher). Blots were then imaged using an iBright FL1000 Imaging System (iBright Firmware version 1.8.1) and quantified using Image J/Fiji (39, 40).

### Mass spectrometry sample collection by immunoprecipitation

HEK293T cells were plated on 15 cm plates. After 24 h of plating, cells were transfected with 15 μg of pCR3.1 plasmids containing C-terminal eGFP-tagged catalytically inactive PA-X D108A or PA-X variants containing both the D108A mutation and the E2 mutations. Transfections were carried out in 10% FBS in DMEM using jetPRIME transfection reagent (Polyplus). eGFP-tagged proteins were isolated from transfected cell lysates using GFP-Trap Magnetic Beads (ChromoTek) following the manufacturer’s protocol. Briefly, 24 h post-transfection, cell lysates were prepared using RIPA buffer (10 mM Tris-HCl, pH 7.5, 150 mM NaCl, 0.5 mM EDTA, 0.1% SDS, 1% Triton X-100, 1% deoxycholate) supplemented with 1× cOmplete EDTA-free protease inhibitor cocktail (Roche), 1 mM PMSF (G-Biosciences), 250 U/mL benzonase nuclease (Sigma-Aldrich), and 2.5 mM MgCl_2_ to ensure complete cell and nuclear lysis. Lysates were then diluted in Dilution buffer (10 mM Tris-HCl, pH 7.5, 150 mM NaCl, 0.5 mM EDTA) supplemented with 1× cOmplete EDTA-free protease inhibitor cocktail (Roche) and 1 mM PMSF. Diluted lysates were added to GFP-Trap Magnetic beads equilibrated in Dilution buffer and rotated end-over-end at 4°C overnight. Beads were washed in Wash buffer (10 mM Tris-HCl, pH 7.5, 150 mM NaCl, 0.05% Nonidet P40 Substitute, 0.5 mM EDTA), and protein was eluted in 200 µL of Acidic elution buffer (200 mM glycine, pH 2.5). The eluate was separated from the beads using a magnet and was neutralized by adding Neutralization buffer (1 M Tris, pH 10.4) to 1/10th of the volume of the Acidic elution buffer.

### Mass spectrometry sample preparation using enzymatic “in liquid” digestion

Immunoprecipitation eluates in Acidic elution buffer (200 µL) were diluted with 360 µL MilliQ-purified water, 140 µL trichloroacetic acid (TCA), and 700 µL acetone (10% TCA and 50% acetone vol:vol final concentration) and incubated on ice for 45 min to precipitate proteins. Precipitated proteins were collected by centrifugation for 10 min at room temperature at 16,000 × *g* and washed twice with cold acetone using the same centrifugation conditions. Protein pellets were air-dried briefly and re-solubilized in reducing buffer (20 μL of 8 M Urea in 50 mM NH_4_HCO_3_, pH 8.5) overnight at 4°C. To reduce disulfide bonds, 1.5 μL of 25 mM dithiothreitol (DTT) in 25 mM NH_4_HCO_3_ (pH 8.5) was added to samples, which were then incubated for 15 min at 56°C. Samples were cooled to room temperature. Cysteines were then alkylated by adding 1.8 μL of 55 mM chloroacetamide and incubating in the dark at room temperature for 15 min. The reaction was quenched with 4.8 μL of 25 mM DTT. Finally, 2.9 μL of lysyl endopeptidase solution (100 ng/μL LysC in 25 mM NH_4_HCO_3_, pH 8.5; FujiFilm) and 9 μL of 25 mM NH_4_HCO_3_ (pH 8.5) were added to a final volume of 40 µL, and samples were incubated for 3 h at 37°C for peptide digestion. The reaction was terminated by acidification with 2.5% trifluoroacetic acid (TFA) added to a 0.3% final concentration. Mass spectrometry sample preparation was carried out by the University of Wisconsin Biotechnology Center Mass Spectrometry Core Facility.

### Nano-liquid chromatography coupled with tandem mass spectrometry

Digests were desalted using Pierce C18 SPE pipette tips (100 µL volume) per the manufacturer’s protocol, eluted in 20 µL of 70/30/0.1% acetonitrile/H_2_O/TFA, dried to completion in a SpeedVac vacuum concentrator, and finally reconstituted in 15 µL of 0.1% formic acid containing 2% acetonitrile. Peptides were analyzed by nano-liquid chromatography coupled with tandem mass spectrometry (nanoLC-MS/MS) using the Agilent 1100 Nanoflow system (Agilent) connected to a hybrid linear ion trap-orbitrap mass spectrometer (LTQ-Orbitrap Elite, Thermo Fisher Scientific) equipped with an EASY-Spray electrospray source (held at constant 45°C). Chromatography of peptides prior to mass spectral analysis was accomplished using a capillary emitter column (PepMap C18, 3 µM, 100 Å, 150 × 0.075 mm, Thermo Fisher Scientific) onto which 3 µL of extracted peptides were automatically loaded. NanoHPLC system delivered solvents A: 0.1% (vol/vol) formic acid, and B: 99.9% (vol/vol) acetonitrile, 0.1% (vol/vol) formic acid at 0.50 µL/min to load the peptides (over a 30-min period) and 0.3 µL/min to elute peptides directly into the nano-electrospray with gradual gradient from 0% (vol/vol) B to 30% (vol/vol) B over 80 min and concluded with 5-min fast gradient from 30% (vol/vol) B to 50% (vol/vol) B at which time a 5-minute flash-out from 50% to 95% (vol/vol) B took place. As peptides eluted from the HPLC column/electrospray source survey, MS scans were acquired in the Orbitrap with a resolution of 120,000, followed by CID-type MS/MS fragmentation of the 30 most intense peptides detected in the MS1 scan from 350 to 1,800 m/z; redundancy was limited by dynamic exclusion. Mass spectrometry analysis was carried out by the University of Wisconsin Biotechnology Center Mass Spectrometry Core Facility.

### Mass spectrometry data analysis

LTQ-Orbitrap Elite-acquired raw MS/MS data files were converted to mgf file format using MSConvert (ProteoWizard: Open Source Software for Rapid Proteomics Tools Development). Resulting mgf files were used to search against user-defined human database (Homo sapiens UP000005640 UniProt reference proteome, 03/08/2024 download containing 82,602 protein entries) plus nine PA-X mutant sequences along with a common Repository of Adventitious Proteins (cRAP), a database of common lab contaminants (116 total entries), using in-house Mascot search engine 3.0.0 (Matrix Science). Carbamidomethylation of cysteines was set as a fixed modification, while N-terminal acetylation, methionine oxidation, and deamidation of asparagine and glutamine were set as variable modifications. Peptide mass tolerance was set at 10 parts per million and fragment mass at 0.6 Da. Peptide and protein annotations, significance of identification, and spectral-based quantification were done with Scaffold software (version 5.0.1, Proteome Software Inc., Portland, OR). Peptide identifications were accepted if they could be established at greater than 90.0% probability to achieve a false discovery rate (FDR) less than 1.0% by the PeptideProphet algorithm (70) with Scaffold delta-mass correction. The ProteinProphet algorithm (71) grouped peptides by their corresponding protein(s). Protein identifications were accepted if they could be established at greater than 99.0% probability by the ProteinProphet algorithm to achieve an FDR less than 1.0% and contained at least two identified peptides. Proteins that contained similar peptides and could not be differentiated based on MS/MS analysis alone were grouped to satisfy the principles of parsimony. Mass spectrometry data analysis was carried out by the University of Wisconsin Biotechnology Center Mass Spectrometry Core Facility.

### 5′ rapid amplification of cDNA ends

RNA was extracted from cells using the Quick-RNA miniprep kit (Zymo Research) following the manufacturer’s protocol and treated with Turbo DNase. After phenol/chloroform extraction from the DNase reaction, the RACE adapter (sequences below) was ligated to 6 μg RNA using T4 RNA ligase (Ambion) at 25°C for 2 h. MMLV RT (Invitrogen) was then used to reverse transcribe ligated RNA to cDNA following the manufacturer’s protocol. Fragments of interest were amplified by Taq DNA polymerase (New England Biolabs) using forward primers annealing to the RACE adapter and reverse primers annealing to the transfected reporter construct (sequences below). PCR products were separated on a 2% agarose gel containing HydraGreen safe DNA dye (ACTGene) and visualized using the iBright FL1000 imager system. DNA was extracted from gel bands at expected sizes and Sanger sequenced to confirm their identities. Primer and adapter sequences are listed in Table 2.

**TABLE 2:** Adapter and primer sequences for 5′ RACE

Name	Sequence (5′→ 3′)
RACE RNA adapter	GCUGAUGGCGAUGAAUGAACACUGCGUUUGCUGGCUUUGAUGAAA
PCR forward primer	GCTGATGGCGATGAATGAACACTG
PCR reverse primer	CCGCCACTGTGCTGGATATCTGCAGAATTCGCGCAAGCAGCAGGGTGTCTATCC

### Quantification and statistical analysis

Data plotted on graphs represent three or more independent biological replicates, as shown by the individual data points included in all graphs. Images shown are also representative of three or more independent biological replicates. Mass spectrometry analyses were carried out on samples collected in two separate experiments. Peptide counts were aggregated and plotted using stacked bar graphs as a percentage of total peptides detected. For box and whisker plots, the boxes extend from the 25th to 75th percentile of values, horizontal lines denote median values, and whiskers are plotted from the minimum to maximum values. Floating bar plots were used instead of box and whisker plots when *n* = 3 (Fig. 3E and 4B), with boxes that extend from the minimum to maximum values and horizontal lines denoting median values. For the scatter plots (Fig. 1E, 2D, 3D, 4D), horizontal lines denote the mean, and error bars denote the standard deviation. For multiple comparisons, one-way or two-way analysis of variance (ANOVA) followed by an appropriate pairwise test corrected for multiple comparisons was used, as indicated in the figure legends. Overall significance was defined as <0.05. Where indicated, levels of significance are denoted as follows: ns = *P* > 0.05, *P < 0.05, **P < 0.01, ***P < 0.001, ****P < 0.0001. Plotting and statistical analyses were performed using GraphPad Prism (v10.5.0). Several experiments were conducted in parallel but are plotted separately in the manuscript. Therefore, in Fig. 1 to 3, control conditions (empty vector, WT PA-X, eGFP, or PA-X D108A) include the same data and are identical, including the representative images shown.

## Data Availability

All data generated during this study are included in this article.
